# Supplementary material for: Entanglement witnesses from mutually unbiased measurements
Source: Sci Rep. 2021 Nov 26;11:22988. doi: 10.1038/s41598-021-02356-2 (PMC8626499; doi:10.1038/s41598-021-02356-2)
Supplement: Supplementary file 1 — Supplementary Information. [file 41598_2021_2356_MOESM1_ESM.pdf]

Entanglement witnesses from mutually unbiased measurements

Katarzyna Siudzińska and Dariusz Chruściński

Institute of Physics, Faculty of Physics, Astronomy and Informatics
Nicolaus Copernicus University, ul. Grudziądzka 5/7, 87–100 Toruń, Poland

October 4, 2021

Appendix A Gell-Mann matrices

Let us take the generalized Gell-Mann matrices defined on $\mathcal{H} \simeq \mathbb{C}^d$ via

$$\sigma_{kl} := \frac{1}{\sqrt{2}} \left(|k\rangle\langle l| + |l\rangle\langle k| \right), \tag{1}$$

$$\sigma_{lk} := \frac{i}{\sqrt{2}} \left(|k\rangle\langle l| - |l\rangle\langle k| \right), \tag{2}$$

$$\sigma_{kk} := \sqrt{\frac{1}{k(k+1)}} \left(\sum_{j=0}^{k-1} |j\rangle\langle j| - k|k\rangle\langle k| \right), \tag{3}$$

where $0 \leq k < l \leq d-1$ and $0 \leq k \leq d-1$, respectively. To construct the mutually unbiased measurements, we group them as in [1],

$$\{G_{\alpha,k} | k = 1, \ldots, d-1\} = \{\sigma_{k,\alpha-1} | k \neq \alpha - 1\},$$
$$\{G_{d+1,k} | k = 1, \ldots, d-1\} = \{\sigma_{kk} | k = 1, \ldots, d-1\}.$$

Then, one has [2]

$$F_0^{(d+1)} = (\sqrt{d}+1) \sum_{l=1}^{d-1} \sigma_{ll}, \tag{4}$$

$$F_k^{(d+1)} = -\sqrt{d}(\sqrt{d}+1)\sigma_{kk} + \sum_{l=1}^{d-1} \sigma_{ll} \tag{5}$$

for $k = 1, \ldots, d-1$, as well as

$$F_k^{(\alpha)} = -\sqrt{d}(\sqrt{d}+1)\sigma_{k,\alpha-1} + \sum_{l \neq \alpha-1} \sigma_{l,\alpha-1}, \tag{6}$$

$$F_{\alpha-1}^{(\alpha)} = (\sqrt{d}+1) \sum_{l \neq \alpha-1} \sigma_{l,\alpha-1} \tag{7}$$

for $k \neq \alpha - 1$ and $\alpha = 1, \ldots, d$.

Appendix B A New Hermitian basis

For the purposes of Example 3, we introduce a new Hermitian operator basis σ'_{kl}. Assume that the operators with only off-diagonal elements are the same as the Gell-Mann matrices; that is,

$$\sigma'_{kl} = \sigma_{kl}, \qquad k \neq l. \tag{8}$$

Now, the diagonal operators are defined as $\sigma'_{00} = \mathbb{I}_d/\sqrt{d}$ and

$$\sigma'_{kk} = \frac{1}{\sqrt{d}(\sqrt{d}+1)}\Big(\mathbb{I}_d + \sqrt{d}|0\rangle\langle 0|\Big) - |k\rangle\langle k|, \qquad k = 1, \ldots, d-1. \tag{9}$$

Obviously, σ'_{kk} are Hermitian, traceless, and together with σ_{kl} they form an operator basis. To check that this basis is indeed orthonormal, it is enough to show that

$$\mathrm{Tr}\sigma'_{kk}\sigma'_{ll} = \frac{2d+2\sqrt{d}}{d(\sqrt{d}+1)^2} + \delta_{kl} - \frac{d}{\sqrt{2}(\sqrt{d}+1)} = \delta_{kl} \tag{10}$$

for $k, l = 1, \ldots, d-1$, as well as

$$\mathrm{Tr}\sigma'_{kk}\sigma'_{lm} = 0 \tag{11}$$

for $k = 1, \ldots, d-1$ and $l, m = 0, \ldots, d-1$, $l \neq m$.

References

[1] A. Kalev and G. Gour. Mutually unbiased measurements in finite dimensions. *New J. Phys.*, 16:053038, 2014.

[2] K. Siudzińska. Generalization of pauli channels through mutually unbiased measurements. *Phys. Rev. A*, 102:032603, 2020.
